# Antiferromagnetism in perfectly ordered L1_0_-MnAl with stoichiometric composition and its mechanism

**DOI:** 10.1038/s41598-020-69538-2

**Published:** 2020-07-27

**Authors:** Suguru Sato, Shuichiro Irie, Yuki Nagamine, Takashi Miyazaki, Yuji Umeda

**Affiliations:** Materials Development Center, Technology and IP HQ, TDK Corporation, Narita, 286-8588 Japan

**Keywords:** Materials science, Condensed-matter physics, Ferromagnetism, Magnetic properties and materials

## Abstract

Manganese (Mn)-based strong magnets have long been a challenge because their 3*d* half-filled nature, owing to the close proximity of Mn atoms, results in antiferromagnetic ordering. Among various Mn magnetic materials, L1_0_-MnAl (τ-phase) has received much attention since it shows ferromagnetism at a high Curie temperature despite the very short Mn–Mn distance. However, because of the difficult synthesis of the stoichiometric and perfectly ordered τ-phase, its intrinsic magnetic properties and mechanism are unclear. Here, we show the first observation of antiferromagnetism, having sixfold magnetic superstructure along the c-axis, in stoichiometric and chemically ordered τ-phase. Moreover, we found that super-exchange interaction between Mn atoms via *p*-electrons of Al atoms causes antiferromagnetism in τ-phase. The ferromagnetism in the conventional Mn-rich τ-phase results from the suppression of the super-exchange interaction due to the substitution the excess Mn atoms for the Al atoms. The current study of Mn-based magnetic materials mainly focuses on the lattice constant engineering based on the simple Beth-Slater picture of direct exchange. These findings present effective ways to obtain high magnetization without antiferromagnetic ordering.

## Introduction

Permanent magnets are essential for advanced green energy technologies such as wind turbines and EV/HEV vehicles. However, the market for permanent magnets mainly consists of Fe- or Co-based magnetic materials. Manganese (Mn) is another inexpensive 3*d* metal that can support high magnetic moments but is not presently a major constituent of permanent magnets. Mn-based magnetic materials potentially surpass these materials because Mn has half-filled d orbitals and thus large magnetic moments^1–3^.

However, the half-filled nature also creates difficulty for the development of Mn-based magnet materials. In order to obtain significant magnetization, a large magnetic moment with ferromagnetic order in a dense-packed structure is needed. Unfortunately, the close proximity of Mn atoms causes direct exchange coupling between Mn atoms, and consequently induces antiferromagnetic ordering due to their nearly half-filled 3*d* orbitals^4,5^. This is the so-called “manganese dilemma”^1^. In general, the Mn atom is antiferromagnetic when the distance of Mn–Mn atoms is less than 2.9 Å. Experimentally, the critical Mn–Mn distance of 2.83 Å has been estimated in NiAs-type Mn compounds^6,7^.

Among various Mn-based magnetic materials, L1_0_-MnAl (τ-phase) has been intensively studied for rare-earth free magnets because of its moderate high-saturated magnetization and high magnetic anisotropy constant^8^. Moreover, from a theoretical point of view, τ-phase is attractive since it shows ferromagnetism with high Curie temperature despite the very short Mn–Mn distance of 2.77 Å. This implies the existence of a mechanism other than direct exchange coupling, which is the origin of the Mn dilemma. Therefore, it is important to clarify the mechanism of ferromagnetism in τ-phase for solving the Mn dilemma.

Experimentally, it is well known that the τ-phase is a metastable phase having off-stoichiometric composition of a Mn content of 55–60 at%^9,10^. The excess Mn atoms occupy Al sites, which leads to antiferromagnetic coupling with the Mn atom in a Mn site^11^, and thus a decrease in the magnetization, which is approximately 1.9 µ_B_/cell^12^. The first principle calculations have predicted that the ideal stoichiometric and perfectly ordered τ-phase shows ferromagnetism with a large magnetic moment of about 2.4 µ_B_/cell^13–16^.

Recently we have reported that a τ-phase with nearly stoichiometric composition with higher-ordering parameter was obtained by post-annealing of electrodeposited MnAl powder, but it unexpectedly showed metamagnetism^17^. However, the mechanism of metamagnetism is unclear, and it may reflect exhibiting antiferromagnetic ground state. Actually, it has been pointed out by Edström et al*.*^18^ that the stoichiometric and perfectly ordered τ-phase shows antiferromagnetism, and the excess Mn atoms essentially contribute to the stability of the ferromagnetic ordering. In other words, the Mn dilemma has been resolved by the excess Mn in τ-phase.

In this paper, we examine the magnetic structure of τ-phase using neutron diffraction experiments and investigate the mechanism of metamagnetism in τ-phase by means of first principle calculation.

## Magnetizations and crystal structures

**Figure 1:**
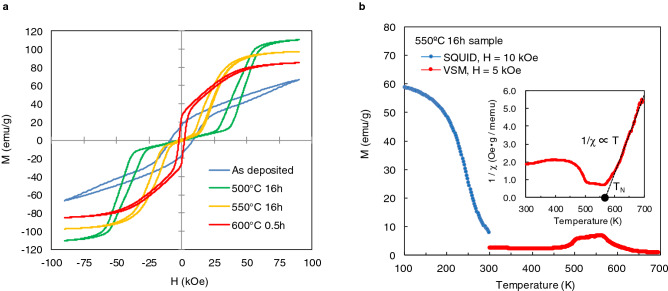
(**a**) Dependence of magnetization curves on annealing condition. Adapted from Fig. 1 in Ref.^17^; licensed under a Creative Commons Attribution (CC BY) license. (**b**), Temperature-dependent magnetizations of the annealed at 550 °C sample. Inset: the temperature dependence of reciprocal susceptibility in high-temperature range 300–700 K. The dotted line is a single straight line fit to the data above 600 K.

The magnetization curves of as-deposited and typical annealing conditions such as 500, 550 °C for 16 h and 600 °C for 0.5 h are shown in Fig. 1a. An as-deposited sample shows ferromagnetic behaviour with a large coercivity of 7.4 kOe. The MnAl powders annealed at 500 and 550 °C shows a sudden increase in magnetization under a magnetic field of approximately 50 and 20 kOe, respectively. This field-induced first-order nonmagnetic to ferromagnetic transition is the so-called metamagnetism. However, the 600 °C-annealed sample shows ferromagnetic behaviour with smaller coercivity than that of the as-deposited one. The magnetism of electrodeposited MnAl powder drastically changes by annealing, which has already been reported in previous article^17^.

For further investigation of the metamagnetic transition, the temperature-dependent magnetizations of the 550 °C-annealed sample were shown in Fig. 1b. In the low-temperature range 100–300 K, the magnetic-field of 10 kOe was applied after zero-field cool. In the high-temperature range 300–700 K, the magnetic-field of 5 kOe was applied. As for the low temperature range, the magnetization gradually increases with decreasing temperature. The magnetization shows a characteristic of the second-order ferromagnetic transition in contrast to a sharp first-order ferromagnetic transition, for example, observed in MnAs^19^. On the other hands, in the range of high temperature range, the magnetization increases as elevating temperature and has a broad peak. This behaviour indicates antiferromagnetic to paramagnetic transition. To clarify this point, the temperature dependence of reciprocal susceptibility in high-temperature range was shown in inset of Fig. 1b. The dotted line, which is a single straight line fit to the data above 600 K, shows the data obeys the Curie–Weiss law and the Neel temperature T_N_ was estimated to be about 570 K. These results indicate the mechanism of metamagnetism involved antiferromagnetic to ferromagnetic transition.

**Figure 2:**
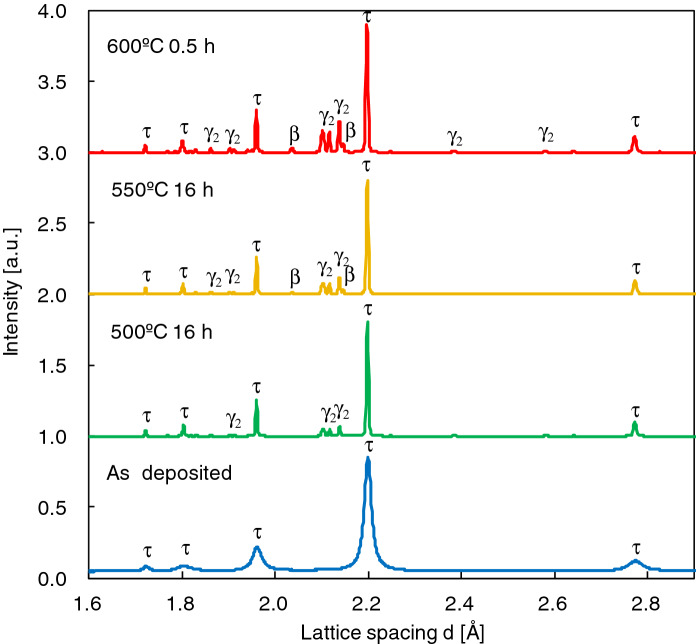
The annealing temperature dependence of SR-XRD patterns.

The annealing temperature dependence of synchrotron radiation X-ray diffraction (SR-XRD) in electrodeposited MnAl powders is shown in Fig. 2. In the as-deposited sample, single-phase τ was observed. As for 400–500 °C for 16 h, only Al-rich Mn_11_Al_15_ (γ_2_-phase)^20^ appeared as the decomposition phase, although the τ-phase is still the main phase. For 550 °C for 16 h and 600 °C for 0.5 h, Mn-rich Mn_3_Al_2_ (β-phase)^21^ was simultaneously observed.

## Magnetic-structure determination by neutron diffraction

**Figure 3:**
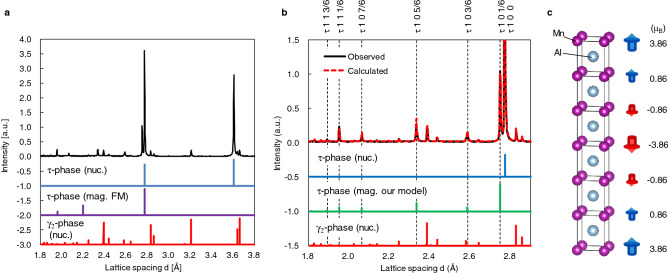
(**a**) Neutron diffraction pattern, (**b**), Rietvelt fitting result, (**c**) the model of magnetic structure of metamagnetic τ-MnAl. The calculated nuclear diffraction patterns of stoichiometric and perfectly ordered τ-phase and γ_2_-phase were indicated as τ-phase (nuc.) and γ_2_-phase (nuc.), respectively. The calculated magnetic diffraction patterns of ferromagnetic and our model of τ-phase were indicated as τ-phase (mag. FM.) and τ-phase (mag. our model.), respectively.

In order to determine the magnetic structure of the τ-phase, neutron diffraction measurement was performed. The neutron diffraction pattern of the 500 °C-annealed sample in the range from 1.8 to 3.8 Å of lattice spacing d is shown in Fig. 3a. Although it is well known that the τ-phase shows ferromagnetism, ferromagnetic peaks (d = 1.9, 2.2 Å) were not observed. However, we found several peaks undetected by XRD, which corresponded to the magnetic scattering indicated by the dashed line in Fig. 3b. These peaks were indexed as rational “l” miller indexes of 1/6, where the unit cell is regarded as a BCT structure. It indicates that there exists a sixfold magnetic superstructure along the c-axis. To investigate the magnetic structure of the τ-phase in detail, the Rietveld fitting was performed. From the results, magnetic moments of Mn direct parallel to the c-axis and the magnitude were 3.86, 0.86, − 0.86, − 3.86, − 0.86 and 0.86 μ_B_, as shown in Fig. 3c. It is noted that the magnitude of the magnetic moment was modulated along the c-axis; this behaviour is the so-called spin density wave (SDW) observed in itinerant electron AFM Cr^22^. Because the sum of these values is zero, we concluded that the magnetic structure of the τ-phase was A-type antiferromagnetic, namely, magnetic moments are anti-ferromagnetically ordered along the c-axis but ferromagnetically ordered in the ab plane. In the ab plane, the existence of strong ferromagnetic exchange interaction has already pointed out by previous theoretical study^2,15^ but the origin of antiferromagnetic ordering along the c-axis is unclear.

Below we discuss the validity of the magnetic moment deduced by our Rietveld fitting. Although the maximal value of the magnetic moment of 3.86 μ_B_ is significantly larger than the theoretical estimation of 2.4 μ_B_^13–16^, if all the magnetic moments are forcedly aligned ferromagnetically, that is, 3.86, 0.86, 0.86, 3.86, 0.86 and 0.86 μ_B_, the average magnetic moment is 1.86 µ_B_/cell, which corresponds to the saturated magnetization of 120 emu/g. Considering the coexistence of non-magnetic decomposition phase, the value is in agreement with the saturated magnetization of 110 emu/g evaluated by using the VSM measurement.

## Rietveld analysis of synchrotron radiation X-ray diffraction patterns

**Figure 4:**
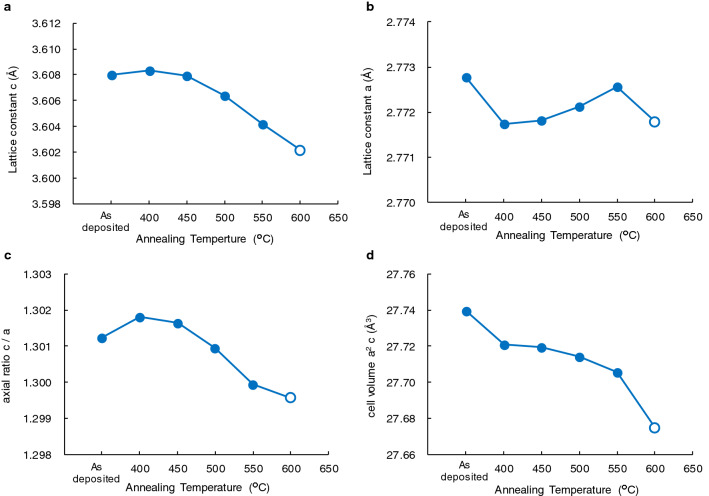
The annealing temperature dependence of (**a**), lattice constant a, (**b**), lattice constant c, (**c**), axial ratio c/a and (**d**), cell volume a^2^c. The values of the samples annealed for 16 h are represented by solid circles, and those annealed for 0.5 h are represented by open circles.

In order to investigate the reason for antiferromagnetism in τ-phase, crystal structural analysis was performed using the SR-XRD. In general, the magnetism of Mn magnetic materials was strongly affected by the Mn–Mn distance. Therefore, the annealing temperature dependence of lattice constants a and c, which are related to the Mn–Mn distance, was estimated using the Rietveld analysis. In the as-deposited sample, the lattice constants were 3.608 and 2.773, respectively. The c value was larger than the value of conventional τ-phase Mn_1.11_Al_0.90_ of 3.57, whereas the a value was equivalent to the conventional value of 2.77^10^. The lattice constant c monotonically decreases above 450 °C, as shown in Fig. 4a. The annealing temperature dependence of the lattice constant a is rather complex, as shown in Fig. 4b. By annealing, the lattice constant a abruptly decreases at 400 °C and gradually increases with the increase in temperature, but it decreases again at 600 °C. Please note that the changes in lattice constants a and c, 0.04 and 0.16%, respectively, were not significantly large.

We also examined the axial ratio c/a and cell volume a^2^c as shown in Fig. 4c,d. The c/a has a peak value at 400 °C and decreases with increase in annealing temperature. As for a^2^c, it monotonically decreases with increase in annealing temperature. It indicates that the Mn composition of τ-phase increases by annealing since the atomic radius of Mn atom is smaller than that of the Al atom. The change in Mn composition might result from decomposition into Al-rich γ_2_-phase, which is expressed as $\uptau \to\uptau +\upgamma _{2}$. This preferential decomposition into Al-rich γ_2_-phase is different from conventional MnAl^23^.

**Figure 5:**
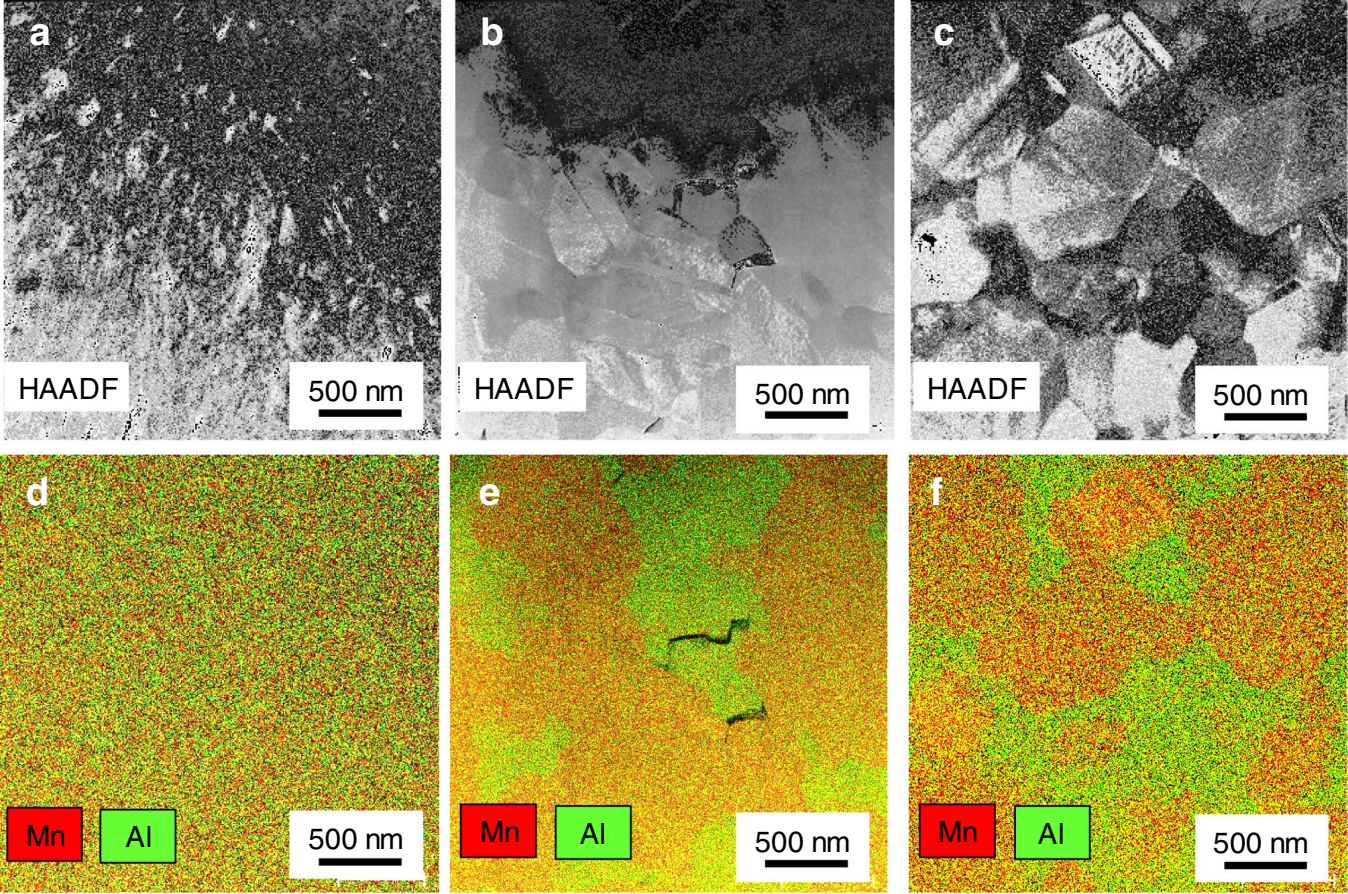
HAADF-STEM imaging of (**a**) as-deposited, annealed at (**b**) 500 °C and (**c**) 600 °C sample. EDS mapping overlay for Mn and Al of of (**d)** as-deposited, annealed at (**e**) 500 °C and (**f**) 600 °C sample acquired by the Bruker Esprit version 1.9 software (https://www.bruker.com/products/x-ray-diffraction-and-elemental-analysis/eds-wds-ebsd-sem-micro-xrf-and-sem-micro-ct/esprit-2.html).

In order to confirm this point, scanning transmission electron microscopy high-angle annular dark-field (STEM-HAADF) imaging and energy-dispersive spectroscopy (EDS) mapping images of as-deposited, annealed sample at 500 °C and 600 °C are shown in Fig. 5a–f. As for as-deposited sample, the spatial distribution of Mn and Al is homogeneous. On the other hands, the annealed at 500 °C and 600 °C samples include both Mn-rich phase and Al-rich phase. By performing selected-area electron diffraction, the Mn-rich and Al-rich phases were identified as τ and γ_2_ phases, respectively. It is noted that the compositional contrast of 600 °C seem to be stronger than one of 500 °C. This point supports the Mn composition change by the decomposition into Al-rich γ_2_-phase indicated by our Rietveld analysis. However, the slight Mn composition change of τ-phase by annealing conditions was not quantitatively demonstrated by our EDS analysis.

**Figure 6:**
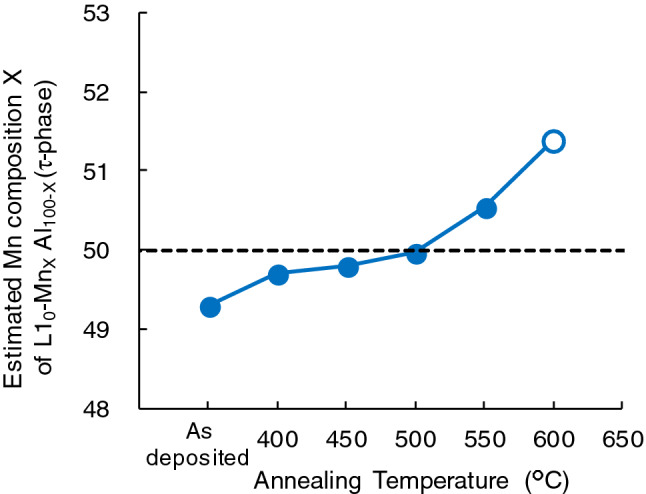
The annealing temperature dependence of estimated Mn composition X of τ-phase. The values of the samples annealed for 16 h are represented by solid circles, and those annealed for 0.5 h are represented by open circles.

In order to investigate quantitatively composition change of τ-phase due to decomposition, the Mn composition x was estimated from the following equation.

$$Mn_{x0} Al_{1 - x0} = \left( {m_{\tau } \times Mn_{x} Al_{1 - x} } \right) + \left( {m_{\gamma 2} \times Mn_{11} Al_{15} } \right) + \left( {m_{\beta } \times Mn_{3} Al_{2} } \right)$$

here m_τ_, m_γ2_, and m_β_ are the molar ratio of each phase, estimated by using the Rietveld quantitative phase analysis. x_0_ is 49.3%, which is the Mn composition of as-deposited sample determined by inductively coupled plasma-atomic emission spectrometer (ICP/AES). The annealing temperature dependence of the Mn composition is shown in Fig. 6. The Mn composition gradually increases with increase in annealing temperature. Note that the stoichiometric composition was realized at 500 °C, where the metamagnetic behaviour is clearly observed.

Although we have already reported^17^, the order parameters S of the τ-phase in the as-deposited samples, the 500 °C annealed sample, and 600 °C annealed sample were 0.76, 0.90 and 0.86, respectively. Here, the ordering parameter S is defined as follows:

$${\text{S}} = 2{\text{g}} - 1$$

where g is the Mn (or Al) occupancy at the Al (or Mn) site of τ-phase. As expected, the ordering parameter of the as-deposited sample increased by annealing. However, the order parameter S of the 500 °C-annealed sample was larger than that of the 600 °C-annealed one. This difference is associated with the increase in Mn composition of the τ-phase accompanied by decomposition. From these results, we conclude nearly stoichiometric and chemically ordered τ-phase having S = 0.90 with Mn = 50.0 at% is obtained by the post-annealing of electrodeposited MnAl powder.

## First principles electronic-structure calculations of L1_0_-MnAl

**Figure 7:**
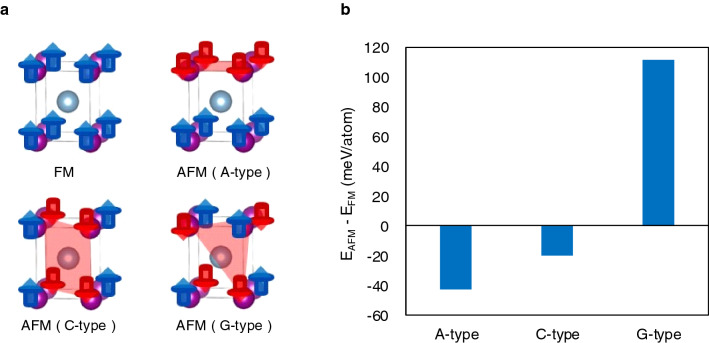
(**a**) Schematic figures for considering magnetic structures in τ-phase and (**b**), calculated total energy differences E_AFM_–E_FM_ between each antiferromagnetic and ferromagnetic structure.

In order to elucidate the mechanism of antiferromagnetism in τ-phase, we investigated the stable magnetic structure by using the first principles calculations. First, we consider the stoichiometric and perfectly ordered L1_0_-Mn_50_Al_50_. We calculated the total energies of the collinear ferromagnetic and antiferromagnetic ordering of the Mn atoms. Possible magnetic structures for L1_0_-MnAl are the collinear ferromagnetic, A-, C- and G-type antiferromagnetic ordering, as shown in Fig. 7a. Comparing the total energies we find that the A-type antiferromagnetic ordering of Mn spins has the lowest energy among the various collinear structures considered as shown in Fig. 7b. These results are consistent with our experimental results as well as with the calculations by A. Edström^18^, although our calculated model was not assumed as a sixfold superstructure along the c-axis. The total energy of the A- and C-type antiferromagnetic ordering was determined to be 42.8 and 19.7 meV/atom lower than that of the ferromagnetic structure. Whereas for the G-type antiferromagnetic ordering, the corresponding value was 111.6 meV/atom higher. The stability trend in antiferromagnetic structures is in agreement with previous studies^16^, except for the result concerning the ferromagnetic state. The magnetic moment of Mn in the A-type antiferromagnetic ordering was 3.21 µ_B_. Although the value is considerably larger than estimations of previous theoretical studies^13,14,18^, it is smaller than the maximal magnetic moment of 3.86 µ_B_ observed by neutron diffraction measurement.

**Figure 8:**
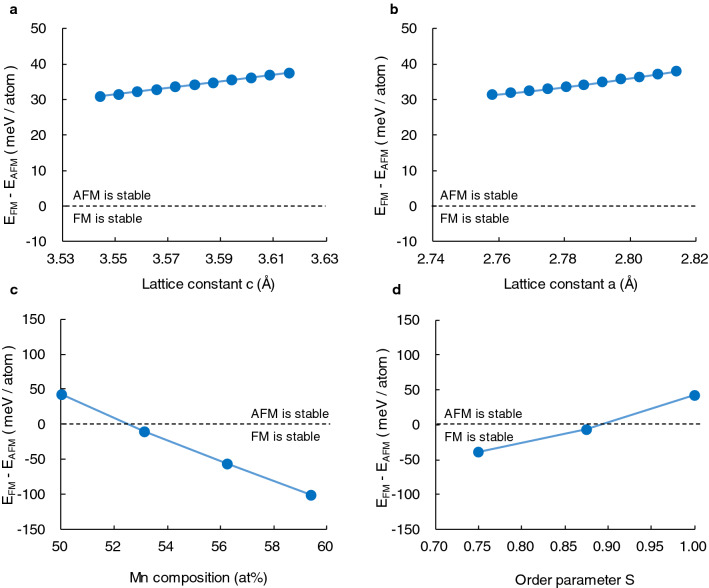
Dependence of (**a**), lattice constant a, and (**b**), c on the total energy difference E_FM_–E_AFM_, between ferromagnetic and A-type antiferromagnetic structure in stoichiometric and perfectly ordered τ-phase. Dependence of (**c**), the Mn composition and (**d**), the ordering parameter on the E_AFM_–E_FM_ where the lattice constant a and c are 2.77 and 3.57 Å, respectively.

If the antiferromagnetism in τ-phase originates from the direct exchange coupling between Mn atoms, the Mn–Mn distance should affect its magnetic structure. Therefore, the dependence of the stable magnetic structure on the lattice constant was examined by first principles calculations without lattice constant relaxation. The dependence of the lattice constant on the stability of ferromagnetism, that is, the total energy difference E_FM_–E_AFM_ between ferromagnetic and A-type antiferromagnetic structure, is shown in Fig. 8a,b. As for the two lattice constants a and c, the antiferromagnetic structure was stable within the lattice constant change of ± 1.0%. Although it seems that the origin of the antiferromagnetism is associated with the Mn dilemma since the lattice constant a, which corresponds to the nearest-neighbour Mn–Mn distance in τ-phase, is smaller than the critical value of 2.9 Å, the ferromagnetic state tended to stabilize as the lattice constant decreased. This implies the existence of an antiferromagnetic mechanism other than direct exchange coupling. Extrapolating these results, the stabilization of the ferromagnetic structure requires a reduction of the lattice constant of more than 10.0%. Therefore, since the lattice constant change by annealing is less than 0.20%, we conclude that the Mn–Mn distance is not the cause of antiferromagnetism in τ-phase.

Our crystal structural analysis revealed the change in Mn composition and the ordering parameter S of τ-phase by annealing. Therefore, we investigate the effect of Mn composition and the ordering parameter S on the stable magnetic structure. The dependence of the Mn composition on the stable magnetic structure was calculated by substituting Mn atoms for Al atoms. On the other hand, the dependence of the order parameter S was calculated by exchanging Mn atoms and Al atoms where the Mn composition is set to 50 at%. In these calculations, there are several non-equivalent configurations due to the arbitrariness of substitution or exchange site. Therefore, the minimum total energy was evaluated by calculating these conceivable non-equivalent configurations. Our calculations find that the antiferromagnetic state is stable for the Mn concentration of 50.0–52.5 at%, but the ferromagnetic state is stable when the Mn concentration over 52.5 at%, as shown in Fig. 8c. This result is consistent with the fact that the conventional Mn-rich τ-phase shows ferromagnetism. This stabilization of ferromagnetism has already been pointed out by previous studies^16,18^. According to S, the antiferromagnetic state is stable as for S > 0.88, as shown in Fig. 8d. These results are quantitatively consistent with showing antiferromagnetism in the 500 °C-annealed sample having S = 0.90 and Mn = 50.0 at% as well as showing ferromagnetism in the as-deposited sample and the 600 °C-annealed sample having S = 0.76, 0.86, respectively. In particular, for the 600 °C-annealed sample, slight Mn-rich composition of 51.7 at% also has contributed to the stabilization of ferromagnetism.

**Figure 9:**
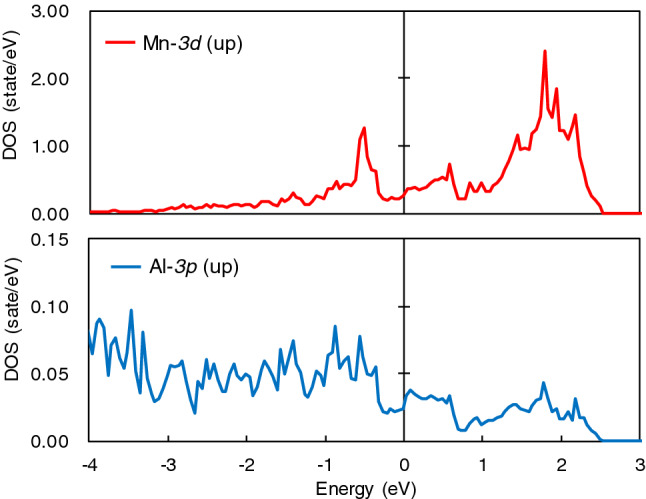
Local partial density of state (LPDOS) of Mn and Al in A-type antiferromagnetic ordering τ-phase MnAl. The horizontal axis is energy, and the vertical axis is density of state.

Finally, we discuss the mechanism of antiferromagnetism in τ-phase. Our calculation clearly showed that the stable magnetic structure in τ-phase is A-type antiferromagnetic and the stable magnetic structure was not strongly affected by the Mn–Mn distance but by Mn occupancy ratio of the Al site, which related to the Mn composition and order parameter S of τ-phase. From these results, it is considered that the origin of antiferromagnetism in τ-phase is super-exchange interaction, which is the magnetic coupling between the magnetic atoms through hybridization with the *p*-orbital electrons of the non-magnetic ligand. In order to confirm the existence of the super-exchange interaction via *p*-electrons of Al atoms, the total energy of hypothetical system Mn_50_Mg_50_ using the same lattice constants of τ-phase was calculated without the lattice and atom position relaxation. As a result, the ferromagnetic structure is preferred over the antiferromagnetic structure in that system. The total energy difference E_AFM_–E_FM_, between A-type antiferromagnetic and ferromagnetic structure, was 55.5 meV/atom. In addition, the results of local partial density of state (LPDOS) of Mn and Al in A-type antiferromagnetic ordering τ-phase MnAl is shown in Fig. 9. The 3*d* orbit of Mn and 3*p* orbit of Al is hybrid above the Fermi energy, as previous reports have suggested^24^. Therefore, we conclude that the mechanism of antiferromagnetism in τ-phase is the super-exchange interaction between Mn atoms via *p*-electrons of Al atoms.

## Conclusions

We conclude nearly stoichiometric and chemically ordered L1_0_-MnAl having S = 0.90 and Mn = 50.0 at% was obtained by the subsequent annealing of the electrodeposited sample, which improves the order parameter S and increases the Mn composition accompanying preferential decomposition into Al-rich γ_2_-phase. Using neutron diffraction, the magnetic structure of the τ-phase was determined to be A-type antiferromagnetic having a sixfold magnetic superstructure along the c-axis. Our calculation clearly showed that the A-type antiferromagnetic state is stabilized when S > 0.88 or Mn < 52.5 at%. In addition, we conclude that the mechanism of antiferromagnetism in τ-phase is the super-exchange interaction between Mn atoms via *p*-electrons of Al atoms, and therefore, the stable magnetic structure was not strongly affected by the Mn–Mn distance but by Mn occupancy of the Al site.

Conventionally, in Mn-based magnetic materials, the Mn–Mn distance has been focused based on the simple Beth-Slater picture of direct-exchange^4^. The Mn dilemma represents the difficulty caused by direct exchange coupling between the Mn atoms. However, this study pointed out the super-exchange interaction of Al electrons via p electrons is dominated in τ-phase. Therefore, the Mn–Mn distance does not strongly affect the magnetism in this system. The mechanism of ferromagnetism in the conventional Mn-rich τ-phase is the suppression of the super-exchange interaction due to the substitution the excess Mn atoms for the Al atoms, which reduces the magnetization simultaneously.

For further improvement of magnetization in τ-phase, it may be necessary to add to elements having no valence *p*-electrons for obtaining high magnetization with ferromagnetic ordering. Furthermore, these findings are considered to be effective for alloys of Mn with elements having p electrons, such as Mn-Ga and Mn-Bi, which also have large potential as a rare-earth free magnets^1,8^.

## Methods

### Sample preparation^17^

The alloy powders consist of τ-MnAl electrodeposited onto planar copper substrates using an aluminium chloride electrolyte composed of a 51:49 mol ratio of AlCl_3_:NaCl containing MnCl_2_ of 1.0 wt%. The electrochemical cell was made of Al_2_O_3_. The cell was heated using a muffle furnace and the temperature was maintained at 300 °C. The counter electrode was an aluminium plate placed parallel to the copper substrate. The alloy powders were electrodeposited galvanostatically at a current density of 40 cm/cm^2^ in an Ar atmosphere. After deposition, the powders were collected by decanting an aluminium chloride molten salt and washing the residual salt in water. The powders were subsequently annealed in an Ar atmosphere.

### Magnetization measurements

Magnetization measurements were carried out using a 9 T physical property measurement system with a VSM manufactured by Quantum Design. Specimens for the VSM measurement were prepared by inserting the powder in a 2 mm × 2 mm columnar-shaped measurement capsule.

The temperature-dependent magnetization in the low-temperature range of 100–300 K were measured using superconducting quantum interference device magnetometer (Quantum Design, MPMS-Evercool). For the measurement, the specimen with paraffin was placed inside a capsule (Eli Lilly, No.4). The specimen was cooled in zero-field down to 100 K. To magnetize the sample, 50 kOe was applied. Then, the field down to 10 kOe field, and the data were recorded up to 300 K. The temperature-dependent magnetization in the high-temperature range of 300–700 K were measured using VSM (Toei Industry, VSM-5). For the measurement, the specimen with quart glass was placed in a copper capsule. Magnetic-field of 5 kOe was applied, and the data were recorded up to 700 K under an Ar flow.

### X-ray diffraction

The XRD measurement for the phase analysis was performed with synchrotron radiation. The measurement was carried out on beam line BL02B2 at SPring-8 in Hyogo, Japan. A specimen was ground to the size of 0.24 × 0.24 × 15 mm and then sealed in a quartz tube capillary filled with an inert gas. The analysis was performed using the Z-Rietveld software^25,26^.

### Microstructural analysis

HAADF and EDS images were obtained using the STEM, Titan-G2 manufactured by FEI. Specimens for the STEM were fabricated by a focused ion beam using Versa 3D manufactured by FEI. STEM-EDS maps were obtained using Super-X EDS detector manufactured by FEI and the Bruker Esprit version 1.9 software (https://www.bruker.com/products/x-ray-diffraction-and-elemental-analysis/eds-wds-ebsd-sem-micro-xrf-and-sem-micro-ct/esprit-2.html).

### Neutron diffraction

Neutron diffraction measurements were performed using the time-of-flight neutron diffractometer iMATERIA (beamline BL20, MLF/J-PARC, Japan). A vanadium cylinder was used as the sample holder. The neutron data were collected at 293 K. The analysis was performed using the Z-Rietveld software^25,26^.

### First principle calculation

The calculations are performed using the frozen-core full-potential projected augmented wave (PAW) method^16^, as implemented in the Vienna ab-initio simulation package (VASP)^27–29^. On-site Coulomb interaction of the d orbitals was corrected using the + U method, where U was determined to reproduce the experimental lattice parameters of MnAl (a = 2.77 Å and c = 3.57 Å). The electronic exchange and correlation effect are described within the generalized-gradient approximation (GGA-PBE_sol_)^30^ to reduce the difference in lattice constant between experimental and calculated one. The energy cutoff of the plane wave basis set is taken as 500 eV. The energy convergence is set at 10^−5^. The supercell was used in the calculations, which consists of 32 atoms (16 Mn atoms and 16 Al atoms). The integrations in the Brillouin zone are performed with k-point grids of 5 × 5 × 5. Unless otherwise noted, the lattice constants, atom position and magnetic moment are relaxed in this study.
